# Basic and clinical immunology – 3034. Peculiarities of parameters immune system in juvenile rheumatoid arthritis

**DOI:** 10.1186/1939-4551-6-S1-P209

**Published:** 2013-04-23

**Authors:** Diloram Abdullaevna Musakhodjaeva, Gulbakhar Kaipbekova

**Affiliations:** 1 Institute of Immunology, Tashkent, Uzbekistan; 2 Republican Scientific and Practical Medical Center of Pediatrics of Uzbekistan, Tashkent, Uzbekistan

## 

Juvenile rheumatoid arthritis (JRA) is one of the most common disabling rheumatic diseases in children in the pathogenesis of which play an important role certain subpopulations of lymphocytes. The functions of these immune cells are associated with the molecular receptors on their CD cellular membranes. The aim of the study was to study the state of molecular receptors of peripheral blood lymphocytes in children with JRA. 32 children aged from 5 to 13 years were examined. Diagnosis is based on the criteria of the American Rheumatology Association. In all patients, the diagnosis was confirmed radiographically. 16 healthy children were in control group. Immunological studies were studying the content of CD3 +, CD4 +, CD8 +, CD16 +, CD25 + and CD95 + cells by monoclonal antibodies. Circulating immune complexes were determined using PEG nephelometry. Phagocytic activity was determined with the use of latex particles. Clinical examination showed that the antibody-positive variant of the disease was observed in 80% of patients seronegative - at 20%. I degree of inflammatory activity was found in 31,1%, II degree - at 53,3%, III degree - in 15.6% of patients. Systemic manifestations were observed in 55.5% of children. Analysis of the results of immunological studies have shown that patients with JRA, compared with those of the control group, there was a reduction of CD3 + (P <0,01), CD4 + and CD8 + (P <0,01). The level of CD16 +-cells was reduced 1.7-fold (P <0,01), while the level of proliferating cells and the number of CD95 +-cells was higher (P <0,01). An increase in the CEC and decreased phagocytic activity of neutrophils. Thus, with JRA identified defects in the immune system as a clear expression of reduction of molecular receptors of T lymphocytes and subpopulation composition, suggesting inferiority of T-cell component of the immune system in children suffering from the disease. Rapid chronization of inflammatory process, autoimmune nature of JRA is probably related to functional deficiency of T lymphocytes.

